# Dynamics of language grounding: on the time course, durability, adaptability, and vulnerability of embodied effects

**DOI:** 10.3389/fpsyg.2025.1637855

**Published:** 2025-09-09

**Authors:** Boris Kogan, Agustina Birba, Mariano Díaz Rivera, Catalina González Santibáñez, Adolfo M. García

**Affiliations:** ^1^Cognitive Neuroscience Center, Universidad de San Andrés, Buenos Aires, Argentina; ^2^Consejo Nacional de Investigaciones Científicas y Técnicas, Buenos Aires, Argentina; ^3^Global Brain Health Institute, University of California, San Francisco, San Francisco, CA, United States; ^4^Departamento de Lingüística y Literatura, Facultad de Humanidades, Universidad de Santiago de Chile, Santiago, Chile; ^5^Escuela de Postgrado, Facultad de Filosofía y Humanidades, Universidad de Chile, Santiago, Chile

**Keywords:** embodied cognition, temporal dynamics, word learning, individual differences, neurodegenerative disorders

## Abstract

From an embodiment stance, semantic processes reactivate specialized brain networks supporting daily experiences. While this general claim has been amply supported, key questions remain unanswered regarding the time course, durability, adaptability, and vulnerability of the underlying mechanisms. This work reviews the main findings on these topics, based on behavioral, neuropsychological, neuroanatomical, hemodynamic, magnetoencephalographic, electroencephalographic, and intracranial methods. The evidence suggests that language-induced sensorimotor reactivations are (a) primary and extended during the temporal flow of meaning, (b) enduring as an anchor for verbal learning throughout life, (c) responsive to individual experiences, and (d) selectively vulnerable to diverse brain alterations. Such conclusions have theoretical, educational, and clinical implications, affording constraints for neurolinguistic models, innovations in language teaching, and early markers of brain disorders. These insights deepen our understanding of the neurocognitive phenomena shaping daily language use.

## 1 Introduction

Several neurolinguistic models propose that semantic processing involves interactions between two (types of) brain systems. On the one hand, multimodal systems, mainly associated with the anterior temporal lobe (Lambon Ralph et al., 2017) and the angular gyrus (Seghier, 2013), are involved in general conceptual processes, regardless of word meaning or the task performed. On the other hand, embodied systems (ES), defined as neural circuits linking sensorimotor activity to modality-specific meaning (Kogan et al., 2020; Pulvermüller, 2005), are differentially activated according to the dominant experiences evoked by the words (Pulvermüller, 2013). For instance, verbs denoting body movements and nouns referring to face parts distinctly engage circuits underpinning action performance (García et al., 2019) and facial recognition (García et al., 2020), respectively. In terms of a prominent account, known as simulation theory, such patterns would indicate that words are understood via partial re-enactment of the experiences they evoke (Gallese and Lakoff, 2005; Glenberg and Kaschak, 2002), contrasting with amodal theories of meaning.

A central mechanism proposed to underpin ES is the mirror neuron system, which supports the coupling between action observation and execution (Rizzolatti and Craighero, 2004; Rizzolatti et al., 2001). Originally described in the premotor cortex of macaques, this system has been implicated in human language processing by virtue of its capacity to map perceived actions onto internal motor representations (Gallese et al., 1996; Gallese and Lakoff, 2005). Building on this foundation, the neural exploitation hypothesis posits that evolutionarily older sensorimotor circuits are repurposed to support abstract cognitive functions, including linguistic meaning (Gallese, 2008; Gallese and Cuccio, 2018). This view has been extended through comparative and neuroscientific work suggesting that the grounding of even abstract concepts may rely on embodied simulation mechanisms (Bonini et al., 2022; Cuccio and Gallese, 2018). These proposals provide a neurobiological basis for the idea that language does not operate independently of the bodily systems anchoring its daily use.

The study of ES has been crucial to the contemporary development of neurosemantics (i.e., the study of the neural basis of meaning). Their characterization challenged mainstream conceptions that reduced conceptual processing to the manipulation of abstract, amodal symbols, construed irrespective brain structure and function (Bedny and Caramazza, 2011). Despite some resistance from modularist views, ES are now widely accepted. Several models focusing on neurocognitive and behavioral aspects of brain-damaged patients (Birba et al., 2017) and healthy subjects (Pulvermüller, 2013; García and Ibáñez, 2016) recognize that language comprehension critically depends on such systems, beyond the role of multimodal systems. However, the study of such systems faces a new agenda marked by questions on their *time course*, *durability*, *adaptability* and *vulnerability*.

First, the time course issue entails a chronometric approach: Is the reactivation of bodily experience, by virtue of ES, a germinal phenomenon during semantic processing, or is it a secondary, epiphenomenal effect? The second question involves an ontogenetic view: Do ES underpin word processing only during infancy, or are they recruited for processing new words throughout life? A third question concerns their experiential adaptability. If ES depend on experience-driven mechanisms, how are they shaped by linguistic competence, athletic performance or the practice of particular tasks? Finally, new questions have arisen regarding their alteration in patients with neurological conditions: Could the disruption of particular brain regions (e.g., motor circuits) cause selective deficits in modality-specific semantic domains (e.g., action verbs)? And if so, what translational avenues can be outlined therefrom?

This work tackles these questions and offers a *dynamic* view of ES in semantic processing. Sections 2 to 5 address each point by integrating behavioral, neuropsychological, neuroanatomical, hemodynamic, electrophysiological, electromagnetic, and neuromodulatory evidence (Table 1), associated with performance in various tasks. Section 6 presents the theoretical and applied implications of these results, outlining relevant challenges. Section 7 synthesizes the main conclusions and highlights their contribution to the understanding of human neurocognition. In sum, this piece reconsiders dynamic aspects of embodied as key tenets of our linguistic and communicative endowment.

**Table 1:** Main types of evidence in the study of embodied systems.

Type of evidence	Main techniques	Key measurements	Major findings*
Behavioral	Behavioral testing	Hits, accuracy, and response times	Bodily movements are affected by the processing of action verbs
Neuropsychological	Clinical and behavioral testing		Patients with movement disorders exhibit distinctive deficits in action verb processing
Neuroanatomical	Magnetic resonance imaging	VBM, SBM, manual lesion tracing	Motor circuit atrophy correlates with impairments during action verb processing
Hemodynamic	Functional magnetic resonance imaging	Blood oxygen level	Various motor regions show peaks of activity during the processing of action verbs
Electrophysiological	Electroencephalography, intracranial recording	Changes in brain electrical activity	Action verb processing involves more functional connectivity between electrodes that are sensitive to motor activity
Electromagnetic	Magnetoencephalography	Magnetic fields produced by brain electrical activity	During the processing of action verbs, the primary motor cortex modulates its activity before multimodal regions
Neuromodulatory	Transcranial magnetic stimulation, transcranial direct current stimulation	Accuracy and response time when stimulating a certain brain region	Stimulation of motor regions selectively influences the processing of action verbs

*Results refer exclusively to the study of action verbs, i.e., linguistic units denoting bodily movements. VBM, voxel-based morphometry; SBM, surface-based morphometry.

## 2 The time course of ES

Research on the time course of embodied reactivations illuminates the temporal microscales at which these occur during semantic processing. The central debate (García et al., 2019; García et al., 2020) is whether embodied phenomena are fast and primary (constitutive of semantic access) or late and secondary (epiphenomenal to other, possibly multimodal, semantic operations).

The proposed cut-off point between primary or epiphenomenal semantic processes is ~200 ms (Pulvermüller, 2018). This threshold follows from principles of electrical propagation between neuronal groups. From word onset, access to sublexical information in the primary auditory or visual cortices occurs in an interval of ~20 to ~90 ms, with partially parallel activation of lexical processes (~100 ms) limited by axonal conduction delay between distant cortical areas (~10–50 ms). Early semantic effects tend to occur only later, in a window of ~120–200 ms (Pulvermuller, 2018). Thus, any semantic effect after 200–250 ms would be post-conceptual, secondary to the inception of meaning proper. In contrast, a semantic effect within ~200 ms (i.e., only ~30–50 ms after lexical access) could hardly be considered post-conceptual (Hauk, 2016). Moreover, if such modulation occurs in a modality-specific area (e.g., primary motor cortex) for words alluding to the same modality (e.g., action verbs), it can be argued that ES play a seminal role in linguistic comprehension.

**Figure 1:**
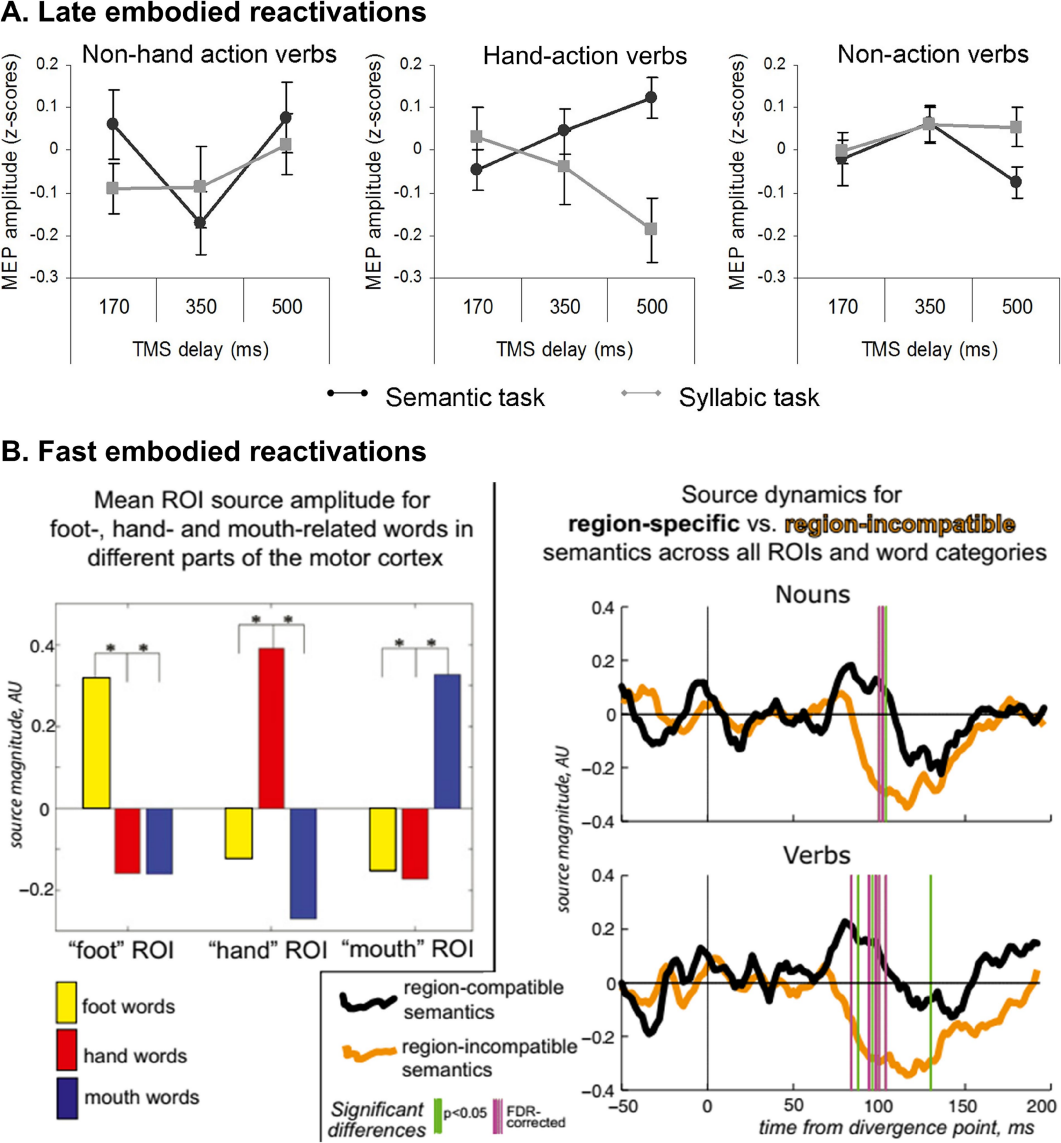
Temporal ubiquity of embodied systems. **(A)** Modulation of motor evoked potentials (MEP) amplitude based on timing delay of primary motor cortex stimulation after verb presentation. Negative modulation of MEP is observed only for the implicit semantic task (syllable counting) on manual action verbs 500 ms after stimulation. **(B)** Semantically specific activation and deactivation of the motor neocortex by action-related words. (Left) ROI-mean peak source activity (z-score normalized for optimal comparison between areas) shows clearly enhanced amplitude for the three word types in each motor ROI. (Right) Pooled source dynamics for activity generated by verbs and nouns in their semantically-specific ROIs as opposed to semantically incongruous ones; vertical bars indicate significant differences. Note not only the early increase of semantic activation for region-specific words starts ∼80 ms after word disambiguation point but also a suppression of source activity for region-incompatible semantics that is maximal slightly later (∼120 ms). Panel **A** is from Papeo et al. (2009). Reproduction authorized under the Creative Commons Attribution license. Panel **B** comes from Shtyrov et al. (2014). Reproduction authorized under the Creative Commons CC BY-NC-ND license.

Several studies have shown that embodied effects can emerge in late time windows. For instance, during explicit (Innocenti et al., 2014) and implicit (Papeo et al., 2009) semantic tasks, motor cortex activity may increase differentially for action verbs when transcranial magnetic stimulation is applied over that region 300 ms and 500 ms after stimulus presentation, respectively (Figure 1, panel A). Likewise, compared to nouns and verbs evoking sensory experiences (e.g., *lighting*, *shine*), those denoting actions (e.g., *spin*, *shake*) involve greater ERP modulations in late time windows (~500 ms) over frontal electrodes associated with motor activity (Barber et al., 2010). This evidence has led some authors to claim that embodied processes cannot be primary. In this sense, Papeo et al. (2009) argue that “the lexical-semantic processing of action verbs does not automatically activate the M1 [primary motor cortex]. This area seems to be rather involved in post-conceptual processing” (p. 1). Even more forceful is the position of (Bedny and Caramazza, 2011), who consider that “understanding the word ‘run’ *occurs* in modality-independent neural systems” (p. 92; our emphasis).

Nevertheless, these judgments seem hasty or at least incomplete. Indeed, the detection of late embodiment effects, with null early effects in a given paradigm, does not exclude the existence of early effects in other tasks. MEG studies consistently yield differential early activation for action-related words in motor regions between ~80 and ~200 ms (Boulenger et al., 2012; Shtyrov et al., 2014; García et al., 2019 Figure 1, panel B). In some cases, such activation patterns even exhibit partially somatotopic distribution –e.g., greater modulation of the hand area of the motor cortex for manual verbs (Pulvermüller, 2018). In the same vein, words referring to sounds (e.g., *bell*) modulate activity in the primary auditory cortex between ~150–200 ms (Kiefer et al., 2008), and negation markers (e.g., *no*) modulate early (~150 ms) markers of motor inhibition (Beltran et al., 2018, see also Montalti et al., 2023 for converging behavioral evidence). Furthermore, electroencephalographic recordings obtained *within* facial processing circuits (such as the right fusiform gyrus) evinced greater modulation for facial nouns (e.g., *nose*) than non-facial nouns (e.g., *arm*) as early as ~100 ms (García et al., 2020).

A leveled view of the evidence, then, indicates that ES can play *both primary and secondary* roles during the emergence of meaning (Harpaintner et al., 2022). In contrast to radical views that attribute the entirety of human comprehension to embodied reactivations (Rizzolatti et al., 2001) and to those who claim that such reactivations are only epiphenomenal (Papeo et al., 2009; Bedny and Caramazza, 2011), we advocate an integrative perspective that recognizes their temporal ubiquity. The simulation of word-induced modality-preferential experiences can be both germinal and post-conceptual during linguistic processing, perhaps depending on stimulus features (Vignali et al., 2023) or task demands (Chen et al., 2013; Harpaintner et al., 2022). This is a clear example of the dynamism of ES in the construction of meaning.

## 3 Ontogenetic durability of ES

A second question concerns the durability of ES. Most evidence in the field comes from participants’ native language (L1). An L1 is present since (and actually before) early childhood, a maturational period that is optimal for incidentally acquiring verbal skills and essential for exploring and developing sensorimotor abilities. In fact, several models support the vital importance of early exposure for new words to become grounded in embodied mechanisms.

However, this does not imply that embodied systems are superfluous for word learning in later life stages. Relevant evidence comes from research on embodied processes in foreign languages (L2) learned after age 7 and in unfamiliar/artificial languages learned by adults (Kogan et al., 2020). Although specific neurocognitive systems (e.g., procedural and declarative mechanisms) are differentially recruited during late L2 compared with L1 processing (Ullman, 2001; Paradis, 2009), lexical units seem to recruit embodied mechanisms irrespective of their age of appropriation.

Several studies show that word action processing in late L2s can interfere with effector-specific movements. For instance, during L2 tasks, manual responses are slower when people process manipulable nouns compared to non-manipulable nouns (Buccino et al., 2017). Likewise, spatial prepositions in L2, such as *über* (over) and *unter* (under) in non-native German speakers, can facilitate congruent upward and downward body movements (Ahlberg et al., 2017; Ahlberg et al., 2018), consistent with ACE-like effects, although the robustness of such paradigms has been subject to recent debate (Morey et al., 2022). Furthermore, during passive L2 reading, motor and somatosensory activation (De Grauwe et al., 2014; Monaco et al., 2023) as well as motor-related cortical activity (Zhang et al., 2024) prove greater for action than for abstract verbs. Since these same effects constitute canonical demonstrations of embodied phenomena in L1 (García and Ibáñez, 2016), these studies suggest that early exposure is not necessary new words to engage modality-specific systems.

**Figure 2:**
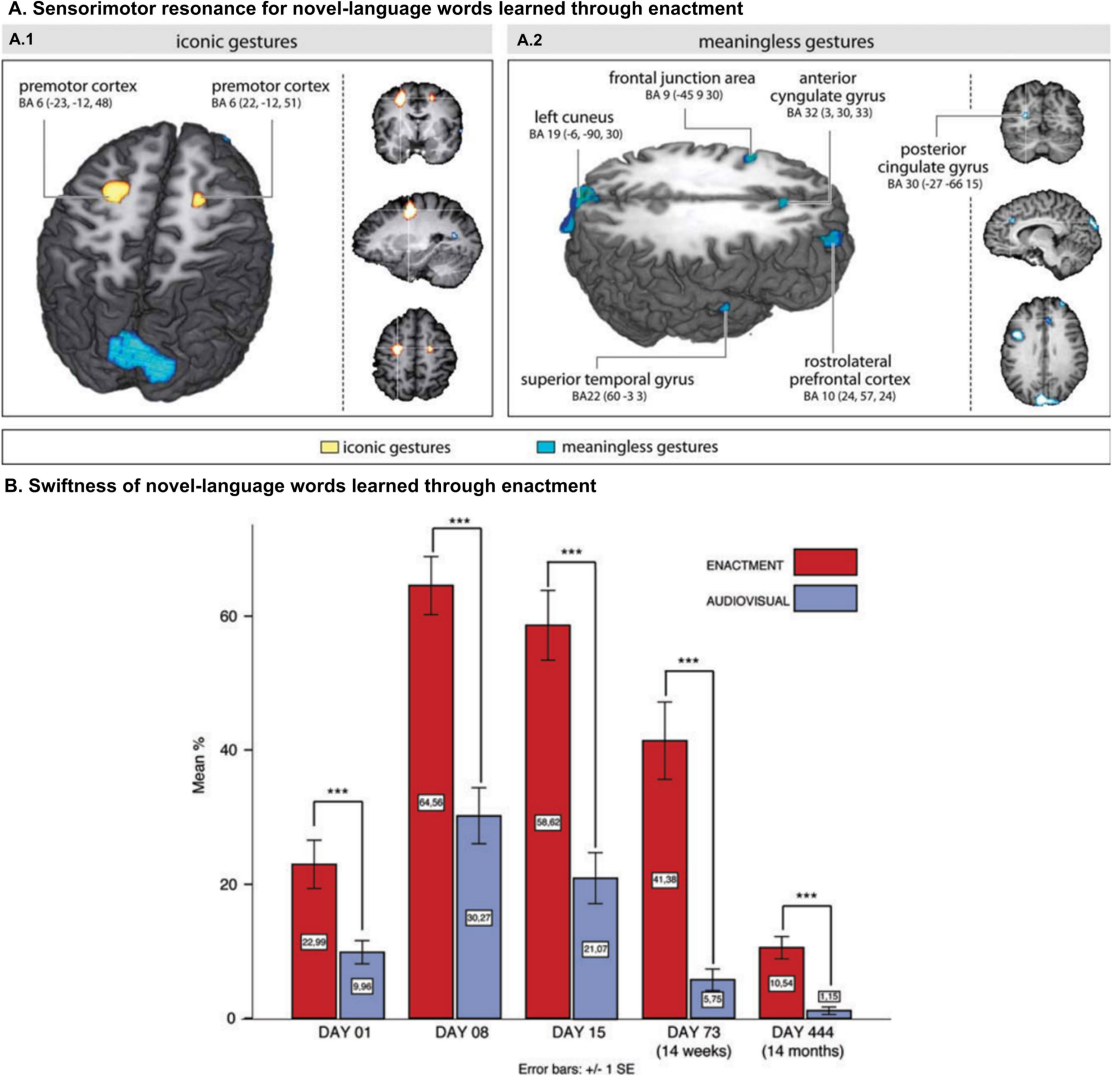
Ontogenetic durability of embodied systems. **(A)** (Left) Main contrast for iconic gestures versus meaningless gestures. Areas of signal intensity change relative to words encoded according to the training conditions, that is, iconic gestures versus meaningless gestures. Motor encoding through iconic gestures elicits activity in the dorsal right and in the left premotor cortices (BA6). (Right) Meaningless gestures create a bilateral large-scale network mirroring cognitive control. The color-coded regions in both figures show clusters with high Bayesian posterior probability of condition. **(B)** Training results for the written translation tests from German into Tessetisch. Words encoded through enactment (EN) are significantly superior in retrieval at all time points. Panel **A** reprinted with permission from Macedonia et al. (2011). Copyright © 2011 Wiley. Panel **B** reprinted with permission from Macedonia and Klimesch (2014). Copyright © 2014 Wiley.

Moreover, ES can be recruited by new words after limited exposure. In fact, adult word learning is enhanced after training with congruent gestures for a few hours over less than a week (Macedonia and Knösche, 2011; Macedonia et al., 2011; Mayer et al., 2015; Macedonia and Mueller, 2016; García-Gámez and Macizo, 2018). Additionally, after such brief exposure, further processing of newly learned words differentially increases activation across motor, premotor, and sensorimotor areas (Macedonia et al., 2011; Mayer et al., 2015; Macedonia and Mueller, 2016; Figure 2, panel A). These fast effects in adults also manifest when new words are incorporated during the observation of third-party actions (Kelly et al., 2009; Freundlieb et al., 2012; Macedonia and Repetto, 2016; Macedonia et al., 2019; Kogan et al., 2020). Of note, such effects are also long-lasting. Lexical consolidation advantages induced by the execution or observation of congruent gestures persist when individuals are retested after 60 (Macedonia and Mueller, 2016), 180 (Mayer et al., 2015), and even 444 (Macedonia and Klimesch, 2014) days (Kogan et al., 2020; Figure 2, panel B).

It seems that both late-incorporated words and L1 words yield similar embodied effects. However, this similarity is not always noticeable. Some neurophysiological and behavioral studies have shown that late L2 embodied effects may be weaker (Vukovic and Shtyrov, 2014; Zhang et al., 2024), less distributed (De Grauwe et al., 2014; but see Tian et al., 2020; Monaco et al., 2023; Britz et al., 2024), only present in highly proficient bilinguals (Bergen et al., 2010; Ibáñez et al., 2010; Vukovic, 2013), and modulated according to the degree of L2 consolidation (Birba et al., 2020; Kogan et al., 2020; Garello et al., 2024; Lu and Yang, 2025).

Also, in the case of bilinguals, late embodied effects might be influenced by interlinguistic dynamics. For instance, Vukovic and Williams (2014) found embodied effects only for homophonous words between L2 and L1 [i.e., those involving sub-lexical overlap between languages, like *cookie* (/kuki/), in English, and *koek* (/kuk/), in Dutch]. The embodied effect induced by spatial prepositions in L2 is also maximized when the L1 employs prepositions with similar spatial associations (Ahlberg et al., 2017; Ahlberg et al., 2018). This suggests that, in some cases, the ES recruited by late-incorporated words might be mediated by the implicit coactivation of lexico-semantic information in L1.

In sum, word learning and processing do not necessarily require early exposure to engage embodied circuits. In fact, such circuits seem to play a key role in learning new terms throughout life. Thus, a dynamic embodied account of language must capture their ontogenetic durability.

## 4 Experiential adaptability of ES

A dynamic perspective of ES must also describe their changes due to individual circumstances. The idea that personal experiences can reconfigure neurolinguistic systems is well documented. For instance, simultaneous interpreters exhibit specific neurophysiological adaptations during translation tasks (Dottori et al., 2020). Similarly, the development of backward speech skills involves anatomical-functional particularities in regions and networks implied in phonological, visual, and domain-general processes (Torres-Prioris et al., 2020). Likewise, it seems that the experiences and situations to which we are continually exposed can shape modality-specific semantic mechanisms.

**Figure 3:**
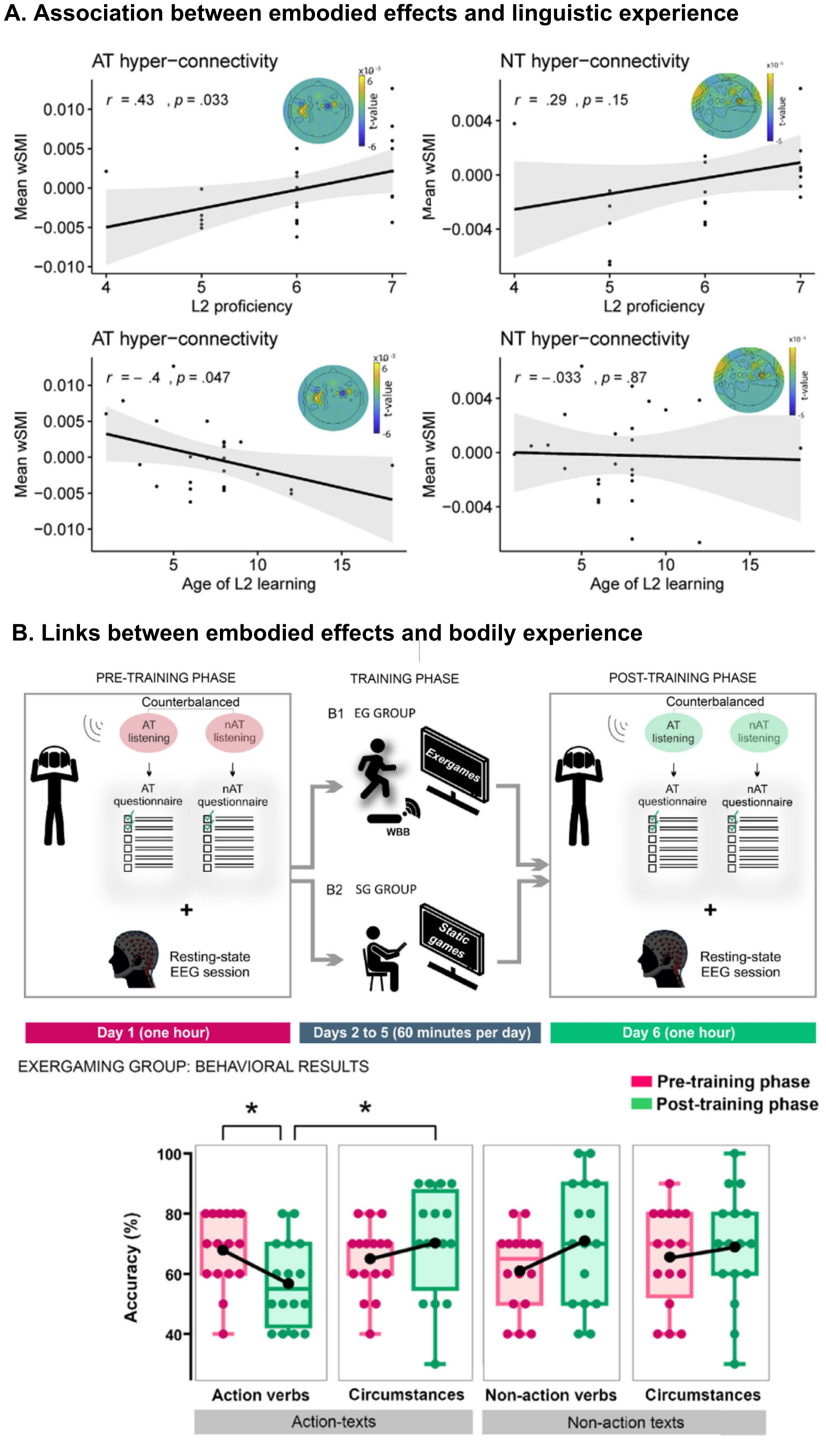
Experiential adaptability of embodied systems. **(A)** Association between foreign language consolidation and embodied phenomena (captured by electroencephalography). Functional connectivity between motor mechanisms when reading action texts in a foreign language positively correlates with second-language proficiency and negatively with age of second-language appropriation. This is not the case when reading neutral texts (without action). **(B)** Pre-training phase: on day 1, subjects first listened to an action text and a non-action text, and answered their corresponding multiple-choice questionnaires (read by the experimenter) after each recording. Then, they sat with eyes closed while EEG activity was recorded at rest. Training phase: from days 2 to 5, subjects completed the videogame intervention using the Nintendo® console. Subjects in the EG group performed an exergaming protocol based on Wii Fit Plus software. Multiple bodily movements were required and captured via a Wiimote and a nunchuck while standing on a balance board. (right) Subjects in the SG group played videogames that required minimal body movements, based on Wii Party software, totally controlled via button presses on the wiimote. Post-training phase: on day 6, subjects first listened to a different pair of action and non-action texts, and answered their respective multiple-choice questionnaires after each recording. Then they completed the same resting-state EEG protocol administered on day 1. The results showed a selective decrease in action comprehension after exergaming. EG, exergaming; SG, static gaming; AT, action text; nAT, non-action text; Pre-T, pre-training; Post-T, post-training. Panel **A** is from Birba et al. (2020). Reproduction authorized under the Creative Commons CC-BY license. Panel **B** is from Cervetto et al. (2022). Copyright (2022), with permission from Elsevier.

First, ES are sensitive to linguistic experience. This has been documented in studies targeting bilinguals with different L2 proficiency levels. For instance, L2 processing of action words (e.g., *clapping*) can slow down congruent limb movements (e.g., hands) and increase the amplitude of the N400 component to incongruent gestures, but only at high proficiency in that language (Kogan et al., 2020). Moreover, when reading L2 action texts, functional connectivity between motor systems increases depending on how early and efficiently that language was incorporated (Birba et al., 2020; Figure 3, panel A). Thus, the degree of language consolidation modulates the level of embodied reactivation during linguistic processing. This sensitivity to linguistic and bodily experience aligns with recent consensus emphasizing that embodied language processing is deeply shaped by individual differences and contextual factors (Ibáñez et al., 2023). Such a perspective highlights the dynamic nature of embodied systems, which continuously adapt to the specificities of the person and environment.

Another relevant factor is bodily experience. Compared to volleyball amateurs and fans, expert players process sport-specific action verbs faster and more accurately (Tomasino et al., 2012), while showing differential activations in left motor and premotor regions (Tomasino et al., 2013). The same happens with hockey experts and fans compared to novice players, a pattern that is accompanied by greater activation of the premotor cortex (Beilock et al., 2008; Yang, 2014). Daily involvement in a given sport, then, seems to attune ES to discipline-specific vocabulary.

Even brief periods of task-specific training can impact on these mechanisms. For instance, repeated transfer of objects between two containers can affect the comprehension of actions involving movements which are congruent with the response direction (Glenberg et al., 2008). Likewise, practicing origami can affect the comprehension of stimuli that evoke congruent movements (Locatelli et al., 2012). Furthermore, during classroom learning of a new language, students who use novel vocabulary in combination with symbolic gestures increase their retention significantly, even 14 months after the lessons (Macedonia and Klimesch, 2014; Kogan et al., 2020). Moreover, repeated use of body-immersive videogames can selectively affect comprehension of actions in naturalistic texts (Trevisan et al., 2017; Cervetto et al., 2022; Figure 3, panel B). Thus, ES are also permeable to brief but focused activities.

In short, linguistic competence, athletic performance, and even brief practice of particular tasks modify the intensity of language-induced sensorimotor reactivations. Accordingly, ES seem to be shaped by our daily activities.

## 5 Selective vulnerability of ES

A fourth topic concerns the vulnerability of ES. Relevant insights come from tests of particular conceptual fields in patients with damage to modality-specific brain circuits. Such alterations in people who once had normal semantic abilities further refine our understanding of ES.

The evidence stems mainly from the study of action language in movement disorders such as Parkinson’s disease (PD) and Huntington’s disease (HD). Both are characterized by atrophy of frontobasal motor circuits, along with primary motor symptoms and other cognitive deficits (Rodriguez-Oroz et al., 2009; Tabrizi et al., 2009). This neurodegenerative pattern has been associated with deficits in the processing of action verbs and concepts, along with alterations in regional activation, functional connectivity, and electrophysiological modulations across motor mechanisms (Birba et al., 2017). Indeed, the greater the atrophy of the basal ganglia (the main structures affected in PD), the greater the recruitment of alternative (non-motor) circuits for processing such verbs (Abrevaya et al., 2017 Figure 4, panels A–C).

**Figure 4:**
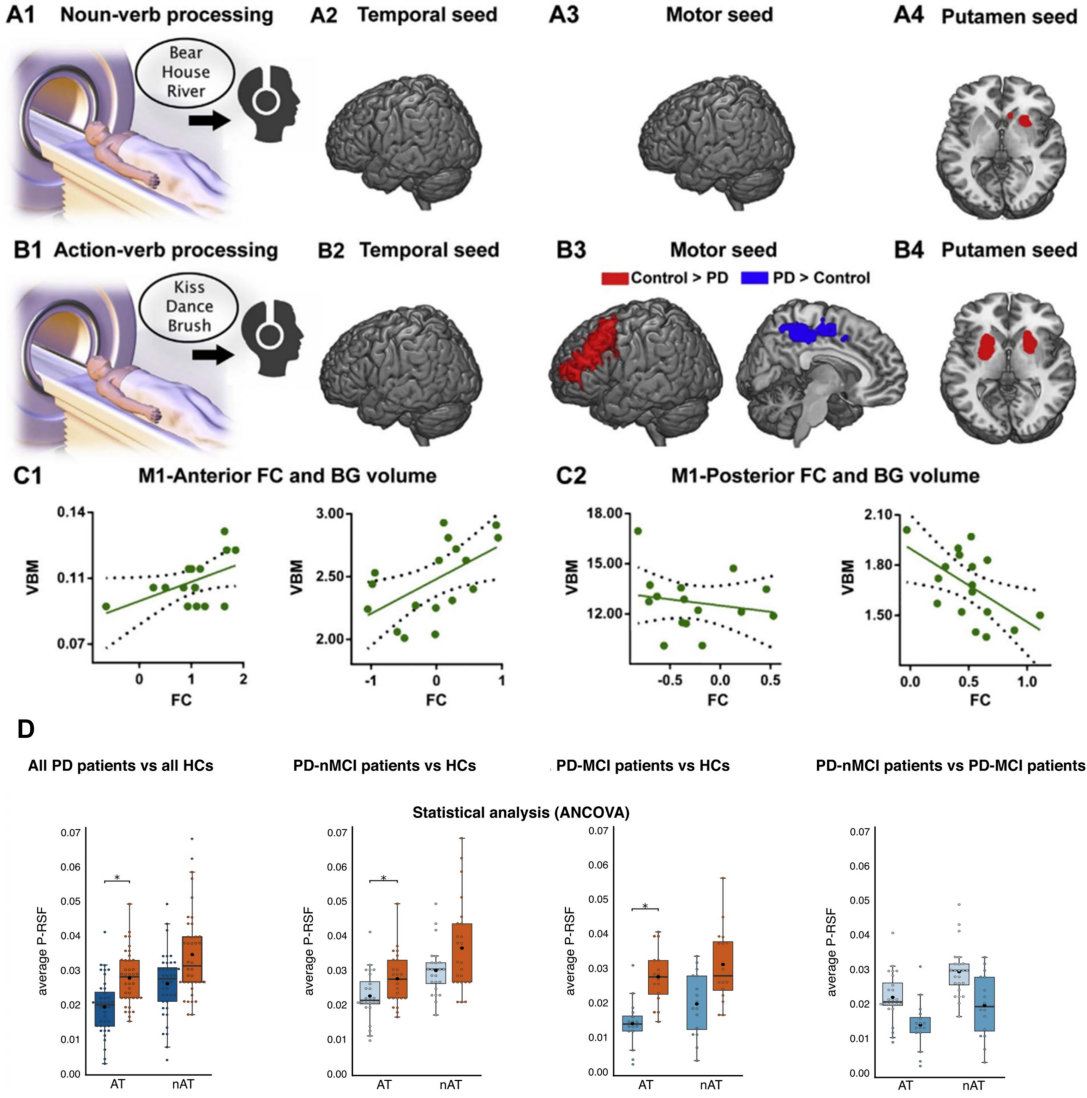
Selective vulnerability of embodied systems. **(A1)** Participants (PD patients and healthy subjects) listened to concrete, non-manipulable nouns inside the scanner. **(A2–A4)** Differences in seed analysis between controls and patients during noun processing. **(B1)** Participants listened to action verbs inside the scanner. **(B2–B4)** Differences in seed analysis between controls and patients during action verb processing. The red color shows significantly higher connectivity cluster (*p* < 0.05) for controls compared to patients. The blue color shows significantly higher (*p* < 0.05) connectivity cluster for patients. **(C1–C2)** Correlations between basal ganglia volume and functional connectivity of the primary motor cortex during action verb processing, for controls and patients. **(D)**. Statistical comparisons between: all PD patients vis-à-vis all HCs, PD-nMCI patients vis-à-vis HCs, PD-MCI patients vis-à-vis HCs and PD-nMCI vis-à-vis PD-MCI patients during action-concept processing. AT action text, nAT non-action text, P-RSF Proximity-to-Reference-Semantic-Field, PD Parkinson’s disease, PD-MCI Parkinson’s disease with mild cognitive impairment, PD-nMCI Parkinson’s disease without mild cognitive impairment. Panels **A–C** are from Abrevaya et al. (2017). Reproduction authorized under the Creative Commons CC-BY license. Panel **D** is from García et al. (2022). Reproduced under the terms of the Creative Commons CC-BY 4.0 license.

These deficits are selective. In lexical decision tasks, PD patients show delays in action verb processing even when they do not exhibit difficulties with abstract verbs (Fernandino et al., 2013). Such patients also show deficits in action verb processing during lexical generation tasks (Péran et al., 2009), with no comparable dysfunctions in other categories. Indeed, the selective impairment for action verbs in this population becomes evident in discursive tasks, both in the productive (García et al., 2016; García et al., 2022) and receptive (Garcia et al., 2018) modality (Figure 4, panel D). This suggests that, in the face of motor system disruption, action language deficits emerge distinctively even in the presence of multiple (con) textual cues.

Such anomalies also disrupt the integration of action concepts with body movements. In healthy persons, the processing of manual action verbs (e.g., *clapping*) affects the execution of hand movements, either delaying or facilitating them (García and Ibáñez, 2016). These semantic-motor integration effects become null in patients with PD and HD, together with aberrant patterns of frontotemporal connectivity (Birba et al., 2017). The same happens in other conditions with motor symptomatology, such as L’hermitte-Duclos disease (Cervetto et al., 2018). In sum, the impairment of ES selectively impacts the ability to integrate verbal meanings with physical movements.

Such deficits come about specifically upon motor system disruptions (they are not caused by just any neurodegenerative condition). For instance, patients with temporo-occipital atrophy (and without motor circuit alterations) exhibit comprehension deficits for nouns but not for action verbs (Steeb et al., 2018). Similarly, the semantic-motor integration effects noted above are preserved in patients with peripheral motor impairments (i.e., not primarily associated with alterations in cerebral motor circuitry), such as neuromyelitis optica and acute transverse myelitis (Cardona et al., 2014). This supports the embodied nature of the deficits referred in PD and HD.

Moreover, in such disorders, action language dysfunctions do not depend on overall cognitive impairment. For instance, in conceptual association (Bocanegra et al., 2015), picture naming (Bocanegra et al., 2017), and textual comprehension (Garcia et al., 2018) tasks, PD patients show specific action semantic deficits, but these difficulties do not depend on patients’ executive or domain-general impairments. This suggests that, when ES are altered, action understanding deficits are *sui generis* –not secondary to other non-specific neurocognitive dysfunctions (Birba et al., 2017; García et al., 2022; Díaz Rivera et al., 2024).

Finally, the selectivity of such deficits is observed in prodromal disease stages. Asymptomatic individuals at genetic risk of developing HD exhibit difficulties with action (but not object) association and abnormal motor-semantic integration (Kargieman et al., 2014). Likewise, in asymptomatic subjects with genetic mutations associated with PD, selective deficits have been documented in embodied linguistic domains (García et al., 2017). Thus, the study of ES could facilitate the detection of individuals at risk of developing PD, HD, or other movement disorders (Palmirotta et al., 2024; Aresta et al., 2025).

In sum, the conceptual domain of action is selectively, specifically, and primarily impaired following motor circuit disruptions. ES can be distinctly altered despite having functioned normally during decades, even when other semantic systems remain unaffected. Therefore, selective vulnerability appears to be another dynamic feature of ES.

## 6 Implications and challenges

The above findings carry several implications. At the theoretical level, three main considerations emerge. First, the debate between strictly multimodal and strictly embodied models is sterile: there is no support for claiming that ES are self-sufficient for semantic processing, nor is there support for describing them as superfluous for such purposes. Our semantic abilities seem to depend jointly on embodied and multimodal systems (among many other mechanisms). Thus, a nuanced account should aim to specify their functional roles and forms of interaction. Second, models that overemphasize early effects over late effects, or vice versa, incur selection biases. Depending on the type of stimulus, task, and dimension of analysis, embodied effects can occur over a wide temporal spectrum starting just past 100 ms and extending beyond 800 ms. The question, then, is not whether ES operate early or late, but rather under what conditions they function more or less rapidly. Third, models that do not explicitly state how ES are shaped by individual experience may promote overly universalistic interpretations of their target phenomena. While any theoretical construct sacrifices particular details to generality, the field has matured enough to incorporate nuances or specifications based on particular subpopulations.

This theoretical perspective is supported by converging neurophysiological and neuroanatomical evidence highlighting a dynamic interaction between embodied and multimodal semantic systems. For instance, García et al. (2019) showed that early embodied effects manifest rapidly within approximately 100 to 200 ms post-stimulus onset, with activations localized in primary motor (M1) and premotor areas, suggesting that sensorimotor reactivations are not epiphenomenal but integral to the initial stages of meaning construction –see also Cervetto et al. (2021). These early embodied activations precede, and likely interact with, later multimodal semantic processing occurring around 300 ms, associated with areas such as the anterior temporal lobe. Similarly, Pulvermüller (2018) articulates a framework of perception-action circuits that support multiple linguistic functions—including memory, prediction, and rule formation—through experience-dependent sensorimotor grounding. This model aligns with the observation that embodied activations occur early and are shaped by individual sensorimotor experiences (Cervetto et al., 2022; Trevisan et al., 2017), thus integrating temporal and topographical data into a comprehensive account. Taken together, these findings emphasize that semantic processing unfolds across multiple temporal and spatial scales, with embodied systems rapidly engaging sensorimotor circuits before interacting with multimodal networks. Such a view cautions against simplistic dichotomies and highlights the necessity of models that explicitly incorporate the influence of personal experience and task context in shaping embodied semantic phenomena.

The evidence also carries educational implications, especially for L2 teaching. Many didactic approaches (such as the audiolingual or the communicative method) have prioritized the combination of oral or written verbal material with pictorial, auditory or audiovisual resources. However, except for particular trends (such as the ‘total physical response’ paradigm), such approaches overlook active bodily experience. Considering the evidence in section 3, it would be worthwhile promoting pedagogical and didactic innovations that integrate embodied approaches to the associative-declarative practices that are usually employed in language teaching.

Specifically, beyond these traditional frameworks, methods that engage learners’ bodily experience more actively—such as drama-based teaching and Total Physical Response—show promising results by fostering stronger links between motor activity and vocabulary learning (Asher, 1969; Cancienne, 2019; Lee et al., 2015; Zhai, 2019). Recent research further refines these approaches by highlighting that learning is optimized when physical actions are effector-congruent with word meanings (García-Gámez and Macizo, 2018; Mayer et al., 2015), and that even brief training can enhance long-term retention (Macedonia and Klimesch, 2014; Macedonia et al., 2011)—indeed, word-learning gains were documented after only 8 h of gesture-word coupling practice. Moreover, novel vocabulary acquisition is boosted through concurrent observation of word-compatible actions, suggesting that embodied language processes benefit even from movement perception (Kelly et al., 2009; Macedonia et al., 2019). Accordingly, embodied strategies could enhance standard declarative practices in language didactics and pedagogy.

Lastly, implications may also be derived for clinical settings. In particular, the results of section 5 could provide sensitive markers for movement disorders, such as PD and HD, as well as amyotrophic lateral sclerosis or spinocerebellar ataxia. Although the data are still incipient and require further replication and validation, embodied alterations in these diseases may be selective (not generalized across language skills in general), partially specific (absent in non-motor disorders), primary (not resulting from global cognitive dysfunctions), linked to critical neurobiological disruptions of such conditions, and potentially detectable in early and even pre-clinical stages. Thus, different linguistic assessments focused on embodied domains could inform clinical practice and enhance diagnostic, prognostic, and monitoring protocols.

Building on these promising findings, screening and diagnostic protocols could be strengthened through language embodiment measures. Such approaches would complement traditional neuropsychological assessments by targeting specific frontostriatal circuit dysfunctions, which often precede overt motor symptoms in disorders like Parkinson’s and Huntington’s disease (García and Ibáñez, 2014; Ross and Tabrizi, 2011). Importantly, embodied language tasks seem sensitive to selective and primary impairments in motor grounding, which may remain undetected by standard cognitive batteries focused on global executive function (García et al., 2016). Moreover, recent advancements in automated naturalistic speech analysis offer scalable and less burdensome assessment tools (García et al., 2025; García et al., 2024), capable of capturing subtle syntactic and semantic deviations linked to embodied processing deficits (Bedi et al., 2014; Eyigoz et al., 2020; García et al., 2016). For example, (García et al., 2022) introduced an automated speech-based metric detecting action-concept impairments that distinguish Parkinson’s patients from controls. The integration of such measures into routine clinical practice holds potential not only for early diagnosis and monitoring of disease progression but also for the development of tailored neurostimulation interventions aimed at modulating compensatory networks (Abrevaya et al., 2017; Tomasino et al., 2014). For instance, (Suárez-García et al., 2021) found that motor cortex neuromodulation selectively improves action-concept processing in PD, independent of general cognitive or motor skills. Consequently, a translational framework that bridges embodied cognitive neuroscience and clinical neurology could significantly enhance both the sensitivity and specificity of language-based biomarkers for movement disorders.

## 7 Conclusion

Research on the semantic role of ES highlights their dynamism across various time scales (from milliseconds to years), neurocognitive dimensions (from behavioral to anatomo-functional manifestations), and personal circumstances (from the development to the loss of specific abilities). These insights can constrain neurolinguistic models, inform language teaching methods, and improve clinical assessment batteries. Moving forward, some of the field’s most pressing challenges involve evaluating, refining, extending, or even falsifying these claims. Whatever the outcome may be, such efforts will help us better understand core aspects of our species’ communicative skills.
